# The Critical Importance of Diagnosing Atypical Hemolytic Uremic Syndrome in Postpartum Renal Dysfunction in a Patient With Systemic Lupus Erythematosus: A Case Report and Comprehensive Review

**DOI:** 10.7759/cureus.78989

**Published:** 2025-02-14

**Authors:** Ayako Inatomi, Shinsuke Tokoro, Daisuke Katsura, Toshihiro Sawai, Takashi Murakami

**Affiliations:** 1 Department of Obstetrics and Gynecology, Shiga University of Medical Science, Otsu, JPN; 2 Department of Pediatrics, Shiga University of Medical Science, Otsu, JPN

**Keywords:** anti-complement monoclonal antibodies, atypical hemolytic uremic syndrome, preeclampsia, systemic lupus erythematosus, thrombotic microangiopathy (tma)

## Abstract

This case report describes a rare instance of a 33-year-old woman with systemic lupus erythematosus (SLE) who experienced a pregnancy complicated by preeclampsia, eclampsia, and postpartum atypical hemolytic uremic syndrome (aHUS). At 28 weeks and four days of gestation, the patient presented with severe hypertension, proteinuria, and a loss of consciousness, leading to an emergency cesarean section. Postoperatively, the patient developed acute kidney injury, respiratory failure, and thrombotic microangiopathy (TMA). Although she exhibited the classic triad of hemolytic anemia, thrombocytopenia, and renal dysfunction, normal complement levels ruled out postpartum exacerbation of SLE, and aHUS was not diagnosed during hospitalization. Differential diagnoses, including HELLP (Hemolysis, Elevated Liver Enzyme levels, and Low Platelet levels) syndrome, thrombotic thrombocytopenic purpura, and Shiga toxin-producing Escherichia coli (STEC)-HUS, were excluded. Schistocytes appeared on postoperative day 5, leading to the cessation of tacrolimus and the initiation of prednisolone. Continuous hemodiafiltration and mechanical ventilation facilitated gradual recovery, and the patient was discharged on postoperative day 26. Post-discharge genetic testing revealed no pathogenic mutations; however, the clinical presentation supported a diagnosis of aHUS. aHUS driven by excessive complement activation requires prompt recognition and treatment with plasma exchange or anti-complement monoclonal antibodies (e.g., eculizumab). In this case, delayed recognition of aHUS precluded the use of such therapies. This case highlights the importance for clinicians to consider the possibility of aHUS in postpartum patients with severe renal dysfunction and TMA symptoms, even if the patient has an underlying SLE, as early diagnosis and treatment of aHUS is necessary to improve maternal outcomes.

## Introduction

Systemic lupus erythematosus (SLE) is a chronic autoimmune disease that affects multiple organs and is particularly common among women of reproductive age [1]. Pregnancy in SLE patients is known to exacerbate disease activity and increase the risk of complications such as miscarriage, preterm birth, preeclampsia, and eclampsia [2]. These complications pose significant risks to both maternal and fetal health, necessitating careful management. Furthermore, atypical hemolytic uremic syndrome (aHUS), a rare but fatal condition caused by complement system abnormalities, can occasionally occur in patients with autoimmune diseases [3,4]. aHUS is characterized by microangiopathic hemolytic anemia, thrombocytopenia, and acute kidney injury, and failure to diagnose and treat it promptly can lead to fatal outcomes. The overlap between SLE and aHUS complicates disease understanding, diagnosis, and the selection of appropriate treatment strategies.

In this report, we describe the case of a 28-week pregnant woman with SLE who developed preeclampsia and eclampsia, necessitating an emergency cesarean section. On postoperative day 2, the patient exhibited respiratory failure and acute kidney injury, requiring continuous hemodiafiltration (CHDF) and mechanical ventilation in the intensive care unit (ICU). Despite severe hemolytic anemia, thrombocytopenia, and acute kidney injury indicative of thrombotic microangiopathy (TMA), the condition was attributed to SLE, and aHUS was not diagnosed during hospitalization. The presence of preeclampsia and eclampsia further complicated the diagnosis of aHUS. It is thus crucial to consider aHUS in postpartum SLE patients presenting with severe renal dysfunction, as this approach is essential for improving maternal prognosis. To the best of our knowledge, this is the first reported case of postpartum aHUS in a pregnancy complicated by SLE. We present this case along with a review of the literature.

## Case presentation

At the age of 23, the patient was diagnosed with SLE based on thrombocytopenia, a butterfly rash, hypocomplementemia, and positivity for anti-dsDNA antibodies. At 25-26 years of age, she tested weakly positive for antiphospholipid (APS) antibodies; however, subsequent tests were negative. Her disease activity was stable on tacrolimus (2 mg) and hydroxychloroquine (200 mg) prior to conception.

At 33 years of age, the patient achieved a natural pregnancy, which was her first. Due to SLE-associated pregnancy, she received prenatal care at a general hospital. Her platelet count ranged between 20,000 and 50,000/μL during early pregnancy, and she was managed with close observation. Aspirin was not prescribed due to thrombocytopenia. At 28 weeks and one day of gestation, her blood pressure began to rise, and her urine protein-to-creatinine ratio increased to 1.34. Home blood pressure monitoring was recommended. At 28 weeks and four days, her blood pressure rose to the 170 mmHg range at home. She experienced a loss of consciousness at home and was transported to our hospital as an emergency. Upon arrival, she complained of severe headaches, had impaired consciousness, and was unable to write. Her blood pressure was in the 160 mmHg range, and her urine protein-to-creatinine ratio had markedly increased to 9.99, consistent with a diagnosis of preeclampsia. Her platelet count was 46,000/μL. However, liver enzyme levels were normal, ruling out HELLP (Hemolysis, Elevated Liver Enzyme levels, and Low Platelet levels) syndrome. Based on the diagnosis of preeclampsia, the episode of loss of consciousness at home was considered highly likely to be an eclamptic seizure. 

On the same day, platelet transfusion was initiated, and an emergency cesarean section was performed under general anesthesia. The neonate was an 1134 g, appropriate-for-date, infant with Apgar scores of 4/9. The umbilical artery blood pH was 7.267, and the base excess was −3.7. Due to prematurity and low birth weight, the neonate was admitted to the neonatal intensive care unit.

**Figure 1:**
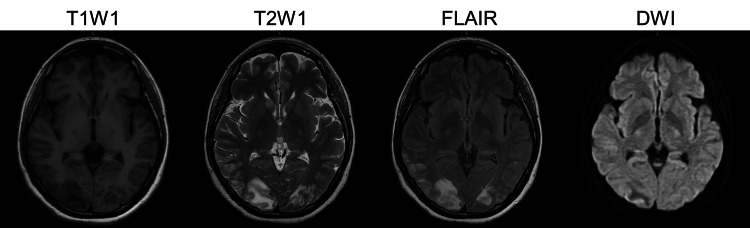
MRI of maternal brain (axial) showing posterior reversible encephalopathy syndrome in a postpartum patient with eclampsia and SLE. T2-weighted, FLAIR, and DWI sequences reveal characteristic hyperintense lesions in the occipital region. T1WI: T1 weighted image; T2WI: T2 weighted image; FLAIR: fluid-attenuated inversion recovery; DWI: diffusion-weighted image.

**Figure 2:**
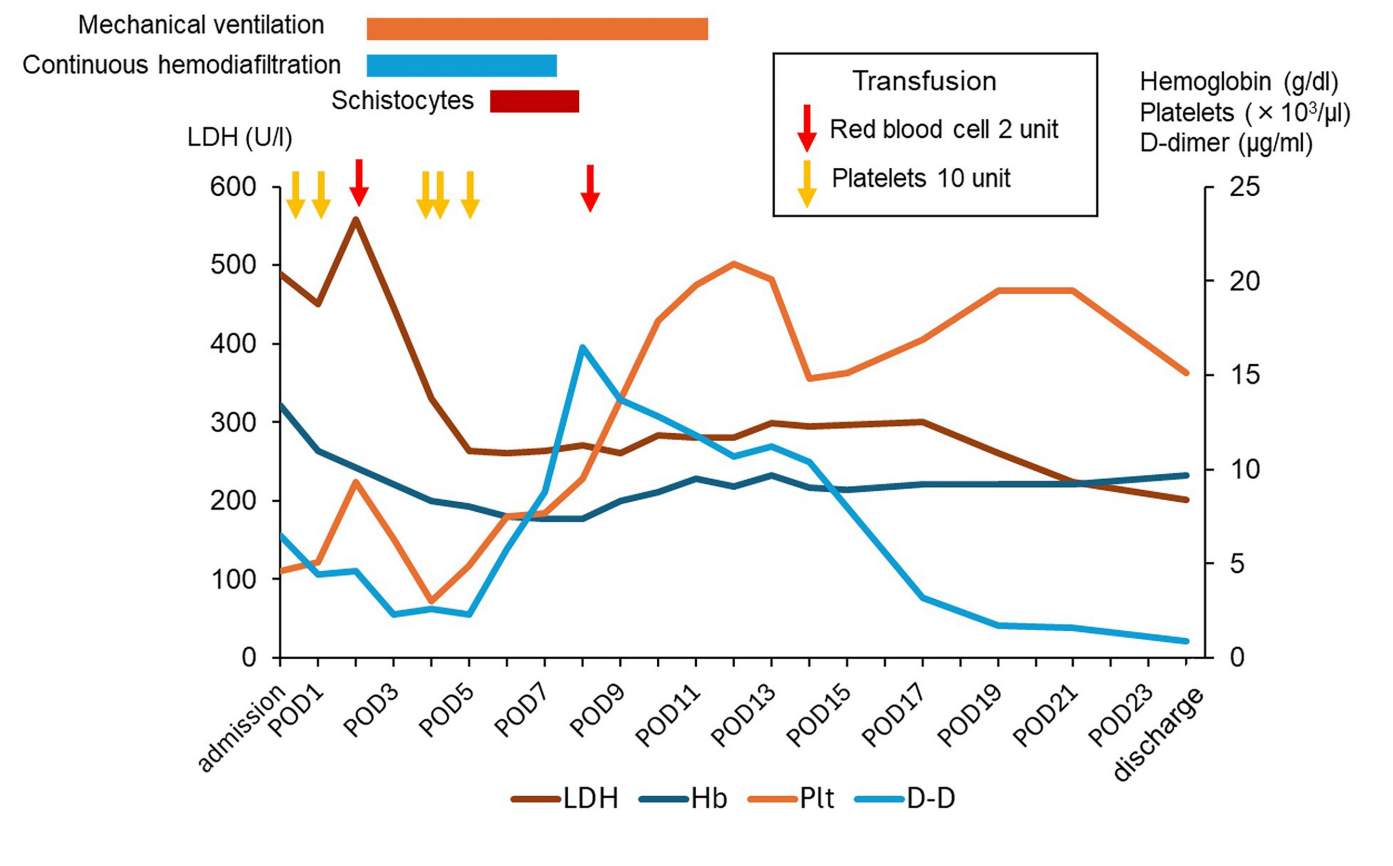
Laboratory parameter trends of lactate dehydrogenase, hemoglobin, platelets, and D-dimer with clinical events from admission to discharge Transfusion events are marked with red (red blood cell transfusion) and yellow (platelet transfusion) arrows, and periods of schistocyte detection are indicated with a red bar. The orange bar represents the period during which the patient was on mechanical ventilation, and the blue bar indicates the period of continuous hemodiafiltration. LDH: lactate dehydrogenase; Hb: hemoglobin; Plt: platelets; D-D: D-dimer

Postoperatively, the mother underwent a head MRI, which revealed posterior reversible encephalopathy syndrome in the occipital region (Figure 1), leading to a diagnosis of eclampsia. On the day of surgery, her urine output was adequate; however, oliguria developed on postoperative day (POD) 1 and progressed to anuria by POD2. Respiratory failure also occurred, necessitating admission to the ICU. On POD2, mechanical ventilation and CHDF were initiated (Figure 2).

Additional red blood cell and platelet transfusions were administered. Complement levels were normal, ruling out postpartum exacerbation of SLE. Repeat tests for APS antibodies and anti-platelet antibodies were negative, as were stool tests for Shiga toxin and enterohemorrhagic *Escherichia coli*.

On POD4, her urine output began to increase, and renal function showed signs of improvement (Table 1).

**Table 1:** Sequential laboratory data from admission to postoperative day 24, highlighting hematological, coagulation, biochemical, and immunological profiles. WBC: white blood cell; RBC: red blood cell; PT: prothrombin time; APTT: activated partial thromboplastin time; AST: aspartate aminotransferase; ALT: alanine aminotransferase; GGT: gamma-glutamyl transpeptidase; LDH: lactate dehydrogenase; BUN: blood urea nitrogen; C3: complement C3; C4: complement C4; NA: not available; POD: postoperative day

Variable	Admission	POD1	POD3	POD5	POD7	POD14	POD24
Blood cell count data							
WBC (×10^3^/μl)	18.6	18.1	15.6	5.4	7.8	10.1	5.6
RBC (×10^6^/μl)	4.49	3.73	3.16	2.71	2.50	2.98	3.35
Hemoglobin (g/dl)	13.4	11.0	9.2	8.0	7.4	9	9.7
Platelets (×10^3^/μl)	4.6	5.1	6.3	4.9	7.7	14.8	15.1
Coagulation data							
PT (s)	10.7	10.4	10.7	10.5	10.7	11.2	10.8
APTT (s)	28.4	30.7	67.4	49	39.5	25.3	25.2
Fibrinogen (mg/dl)	434	403	376	524	420	NA	NA
D-dimer (μg/ml)	6.5	4.4	2.3	2.3	8.8	10.4	0.9
Chemical data							
AST (U/l)	21	19	22	25	25	31	17
ALT (U/l)	7	6	8	15	17	71	27
GGT (U/l)	18	14	18	74	337	369	198
Total bilirubin (mg/dl)	0.8	0.5	0.5	0.5	0.6	0.6	0.6
LDH (U/l)	489	450	447	264	263	294	201
BUN (mg/dl)	14.7	15.3	24.4	6.4	13.7	23.9	13.8
Creatinine (mg/dl)	0.83	0.75	1.28	0.42	0.65	0.34	0.48
Sodium (mmol/l)	136	137	132	136	141	142	142
Potassium (mmol/l)	3.6	3.4	4.1	4.4	3.9	3.4	3.4
C3 (mg/dl)	94	NA	85	100	138	NA	NA
C4 (mg/dl)	11	NA	19	23	32	NA	NA

Schistocytes appeared on POD5. Thrombotic thrombocytopenic purpura (TTP) was suspected, and ADAMTS13 activity was measured; however, no significant decrease was observed (60%), and ADAMTS13 inhibitors were negative. Tacrolimus was discontinued on POD6 due to the presence of schistocytes, and oral prednisolone (30 mg) was initiated. CHDF was discontinued on POD6 as her urine output increased. The patient was extubated on POD11 and discharged on POD26. At discharge, her medications included prednisolone (20 mg), amlodipine (10 mg), telmisartan (80 mg), and hydroxychloroquine (200 mg).

The severity of thrombocytopenia and renal dysfunction had peaked on POD3, and the appearance of schistocytes on POD5 deviated significantly from the typical clinical course of preeclampsia, which usually resolves postpartum. These atypical features led to a suspicion of aHUS after discharge. Two months postoperatively, the patient underwent genetic counseling and genetic testing for aHUS. Although no genetic mutations were identified, she was clinically diagnosed with aHUS.

## Discussion

This case represents a rare instance of pregnancy complicated by SLE that progressed to preeclampsia, eclampsia, and subsequently aHUS. Despite exhibiting the triad of hemolytic anemia, thrombocytopenia, and renal dysfunction, and with normal complement levels ruling out postpartum exacerbation of SLE, the complex clinical presentation prevented the diagnosis of aHUS during hospitalization. The objective of this report is to examine the pathophysiological relationship between SLE, pregnancy-related hypertensive disorders, and aHUS, and to derive lessons for clinical management.

There are several differential diagnoses to consider in pregnant and postpartum patients with thrombocytopenia. In this case, it was crucial to differentiate between HELLP syndrome, exacerbation of SLE, Shiga toxin-producing *E. coli* (STEC)-HUS, TTP, and secondary TMA [5].

HELLP syndrome is a severe complication of hypertensive disorders of pregnancy characterized by hemolysis, elevated liver enzymes, and low platelets. It predominantly occurs in late pregnancy or shortly postpartum and presents with symptoms such as right upper quadrant pain, nausea, headache, and hypertension. Widely used diagnostic criteria include those proposed by Sibai in the Tennessee classification: (1) hemolysis (abnormal red cell morphology on a peripheral smear, bilirubin ≥1.2 mg/dL, and lactate dehydrogenase (LDH) >600 U/L), (2) liver dysfunction (aspartate aminotransferase (AST) ≥70 U/L), and (3) thrombocytopenia (platelet count <100,000/μL), with all three criteria required for diagnosis [6]. In this case, while thrombocytopenia was present, liver enzyme levels were normal, ruling out HELLP syndrome. Additionally, the worsening of TMA after delivery further ruled out HELLP syndrome, as it typically resolves postpartum [7].

Next, the absence of SLE exacerbation was confirmed. SLE primarily affects women of childbearing age. Its etiology involves genetic predisposition, environmental triggers, and immune dysregulation, leading to systemic inflammation and multi-organ involvement [8]. Evaluation of SLE exacerbation requires assessment of complement levels. In this case, normal complement levels ruled out postpartum exacerbation of SLE. While APS can contribute to thrombocytopenia in SLE patients [9], the patient had tested negative for APS antibodies before pregnancy, and repeat testing during ICU admission was also negative.

The possibility of STEC-HUS and TTP was also investigated. STEC-HUS is a TMA caused by uncontrolled complement activation or direct cellular injury induced by Shiga toxin produced by certain bacterial strains, particularly *E. coli *[10]. In the current case, stool culture and testing for Shiga toxin confirmed the absence of STEC-HUS. TTP is a TMA caused by significantly reduced ADAMTS13 activity, leading to unchecked von Willebrand factor-mediated platelet aggregation [11]. In the current case, ADAMTS13 activity was within normal limits, and no ADAMTS13 inhibitors were detected, ruling out TTP. Additionally, as the patient had been taking tacrolimus, the possibility of drug-induced secondary TMA was considered, and tacrolimus was discontinued postpartum.

Based on the clinical course and the exclusion of other conditions, a final diagnosis of aHUS was made. aHUS is characterized by uncontrolled and excessive complement activation, leading to endothelial cell injury and TMA. This condition results in severe organ damage, end-stage renal failure, and even death. Mechanisms of sustained complement activation in aHUS include genetic mutations in *C3, CFB, CFH, CFI, MCP*, and *THBD*, genetic polymorphisms in *CFH* and *CFHR1*, and autoantibodies against complement factor H (CFH) [4,12]. A definitive diagnosis of aHUS is established by identifying pathogenic genetic variants or anti-CFH antibodies. However, pathogenic variants are reported in only 40-60% of aHUS cases, and their absence does not rule out the condition [13]. In a study of pregnancy-associated aHUS, genetic mutations were identified in five of seven patients (71%) [14]. In the current case, while no genetic mutations were identified, a clinical diagnosis of aHUS was made. The presence of SLE in this patient suggests an underlying immune dysregulation, which predisposes to complement abnormalities and increases the risk of aHUS. Furthermore, pregnancy itself is a known trigger for complement activation [7], which likely contributed to the onset of aHUS in this case. Additionally, eclamptic seizures are strongly suspected as a contributing factor to disease onset. Providing patients and primary care physicians with appropriate information is essential to ensuring early diagnosis and prompt treatment when aHUS recurs. An increase in sC5b-9 has also been reported as a supportive diagnostic marker for aHUS [15]. Elevated sC5b-9 indicates complement activation and may support the diagnosis of aHUS; however, it can also be elevated in other TMAs and is not a specific marker. Therefore, the utility of sC5b-9 should be considered in conjunction with clinical and laboratory findings rather than as a standalone marker.

Regarding treatment, the standard therapeutic options for aHUS include plasma exchange and anti-complement (C5) monoclonal antibody agents such as eculizumab and ravulizumab [4,12]. Anti-complement (C5) monoclonal antibodies have the potential to improve the condition by directly controlling abnormalities in the complement system, and their efficacy has been increasingly reported in recent years. Early initiation of eculizumab has been suggested to improve renal function in aHUS patients and reduce the need for long-term dialysis. On the other hand, as eculizumab inhibits the terminal complement pathway, it significantly increases the risk of meningococcal infections. Patients should receive meningococcal vaccination before initiating treatment, and prophylactic antibiotics are often recommended during therapy. Additionally, eculizumab is expensive and may be difficult to access in certain regions. In this case, the introduction of complement inhibitors should have been considered as a treatment option once schistocytes appeared and TTP was ruled out. Had complement inhibitors been administered, renal dysfunction might have improved earlier, potentially leading to a shorter hospitalization period. However, the rapid postoperative progression and the complexity of the patient’s condition limited the available therapeutic options.

## Conclusions

Although rare, aHUS should be considered in SLE pregnancies presenting with severe renal dysfunction, thrombocytopenia, and hemolytic anemia. Early recognition and appropriate treatment of aHUS are essential to improving the long-term prognosis of patients.
